# Time-Dependent Electrochemical Behaviour and Surface Response of Ti6Al7Nb and Ti6Al4V Alloys in Albumin-Containing PBS

**DOI:** 10.3390/ma19101929

**Published:** 2026-05-08

**Authors:** Senka Gudić, Aleš Nagode, Ladislav Vrsalović, Jakov Meštrović, Luka Marijan Alešković, Ivana Smoljko

**Affiliations:** 1 Faculty of Chemistry and Technology, University of Split, 21000 Split, Croatia; ladislav.vrsalovic@ktf-split.hr (L.V.); luka@aleskovic.com (L.M.A.); ivana.smoljko@ktf-split.hr (I.S.); 2 Faculty of Natural Science and Engineering, University of Ljubljana, 1000 Ljubljana, Slovenia; ales.nagode@ntf.uni-lj.si; 3 Faculty of Health Sciences, University of Split, 21000 Split, Croatia; jakov.mestrovic@ozs.unist.hr; 4 School of Medicine, University of Split, 21000 Split, Croatia

**Keywords:** titanium alloys, cp-Ti, Ti6Al7Nb, Ti6Al4V, electrochemical behaviour, corrosion, albumin, protein adsorption, passive film, biomaterials

## Abstract

The electrochemical and surface behaviour of implant-grade commercially pure titanium (cp-Ti), Ti6Al7Nb, and Ti6Al4V clinically used alloys was investigated in phosphate-buffered saline (PBS), with and without albumin, at 37 °C using electrochemical techniques and SEM/EDS analysis. In PBS, the corrosion resistance followed the order Ti6Al4V < Ti6Al7Nb < cp-Ti, with cp-Ti serving as the benchmark reference material. The addition of albumin improved the corrosion resistance of all materials under the investigated static in vitro conditions, with the most pronounced effect observed for Ti6Al4V. Over time, the oxide layer stabilised and the differences between the materials decreased, with Ti6Al4V approaching the behaviour of more stable systems. Surface analysis revealed a comparatively more uniform (but not fully homogeneous) protein distribution on Ti6Al7Nb, whereas Ti6Al4V exhibited localised adsorption associated with greater surface heterogeneity. Protein adsorption acts as a stabilising interfacial factor, contributing to improved protective properties under static conditions. These findings highlight the key role of surface heterogeneity in governing protein adsorption and electrochemical behaviour of titanium alloys under biologically relevant conditions.

## 1. Introduction

Biomedical devices are predominantly made from metallic materials (70–80%), which are exposed to aggressive body fluids such as saliva, blood, and synovial fluid [1]. These fluids are considered aggressive due to their relatively high concentrations of chloride and other ions, the presence of oxygen, and variations in pH and temperature. Biocompatible materials, particularly titanium and its alloys, have played a key role in medicine and dentistry for decades in the manufacture of various implants. They are most commonly used in orthopaedics and dentistry, but also in applications such as vascular stents, heart valves, pacemakers, and artificial hearts [2]. Their widespread use is based on excellent corrosion resistance, mechanical strength, and good compatibility with biological tissues [1,2,3,4,5,6]. Commercially pure titanium (cp-Ti) was included in this study as a benchmark material, as it represents the clinically established standard for corrosion-resistant biomaterials, allowing direct comparison between alloyed systems and the baseline passive behaviour of titanium. The materials studied (cp-Ti, Ti6Al7Nb, and Ti6Al4V) belong to a group of alloys with extensive and long-term clinical applications, highlighting the relevance of this study. However, it is important to distinguish their behaviour in real, dynamic biological systems from that in simplified static in vitro conditions, which are necessary to understand the underlying mechanisms.

The key factor enabling these properties is naturally formed oxide layer (TiO_2_), only a few nanometres thick, which acts as a protective barrier between the implant and the biological environment [6,7,8,9]. The stability and longevity of implants largely depend on this layer, whose protective efficiency is governed by its thickness, structure, and the microstructure of the substrate [10,11]. Corrosion behaviour under physiological conditions depends not only on intrinsic material properties, but also on the composition of the surrounding electrolyte.

Albumin is the most abundant protein in blood plasma and is primarily responsible for forming the so-called “protein corona” on biomaterial surfaces [12,13,14,15,16]. Numerous experimental studies over the past decade have shown that the addition of proteins to the test medium can significantly affect key electrochemical parameters of titanium and its alloys. Upon adsorption onto the implant surface, albumin can block active sites and defects in the oxide layer, alter surface energy, and promote the formation of a more stable, compact protective layer, thereby reducing ion diffusion and enhancing the material’s protective properties under specific environmental conditions [17,18,19].

For example, Radovanović et al. reported that the addition of collagen and albumin to simulated body fluids enhances the corrosion performance of titanium, as indicated by increased impedance and reduced corrosion current density [17]. Similar beneficial effects of albumin have been observed in albumin-containing media for other titanium alloy systems. Nartita et al. [20] reported that albumin improves the corrosion behaviour of Ti–Zr alloys, while Sotniczuk et al. [21] demonstrated that albumin stabilises the oxide layer and reduces its defectiveness on Ti–Nb alloys.

However, the influence of proteins on corrosion behaviour is not unequivocal and depends strongly on environmental conditions. For instance, in the presence of proinflammatory species such as hydrogen peroxide, increased dissolution of Ti6Al4V has been reported, associated with changes in electrochemical potential and protein–metal interactions [22,23,24,25]. In addition, different titanium alloys do not respond equally to protein-containing environments. Hrir et al. [26] showed that certain α-Ti alloys exhibit improved behaviour under these conditions, whereas Ti6Al4V may be more susceptible to local destabilisation in more aggressive media. Recent studies have also reported improved electrochemical corrosion performance of novel β-type titanium alloys in protein-containing PBS media compared with conventional Ti6Al4V and cp-Ti systems, highlighting the ongoing development of advanced biomaterials with enhanced corrosion stability under physiological conditions [27]. Nevertheless, despite these advances, clinically established alloys such as Ti6Al7Nb and Ti6Al4V remain highly relevant because of their widespread long-term medical use and well-documented clinical performance.

Furthermore, the corrosion behaviour of titanium alloys is strongly influenced by their composition and microstructure. Substitution of vanadium with niobium has been associated with enhanced stability due to the formation of more stable oxides (TiO_2_ and Nb_2_O_5_) and a more favorable microstructure [28]. Recent studies further confirm that both microstructure and chemical composition play a key role in the formation and durability of passive films in chloride-containing environments [29].

Despite numerous studies, a systematic understanding of the long-term effects of protein exposure on the electrochemical behaviour of titanium alloys remains limited, particularly regarding differences between materials with varying microstructures and compositions. In particular, few studies simultaneously address free corrosion over extended periods, anodic behaviour in protein-containing environments, and direct comparisons of clinically relevant materials under identical conditions.

This work investigates the influence of albumin on the electrochemical behaviour and stability of oxide layers on commercially pure titanium (cp-Ti) and the Ti6Al7Nb and Ti6Al4V alloys in physiologically relevant PBS. The study focuses on time-dependent changes and differences between materials, combining long-term monitoring of free corrosion with anodic testing in the presence of proteins, and correlating electrochemical responses with surface morphology and chemical composition.

## 2. Materials and Methods

### 2.1. Materials

The study was conducted on cp-Ti (commercially pure titanium, Grade 2), Ti6Al7Nb, and Ti6Al4V—materials particularly relevant for medical implants. The materials were supplied as commercially manufactured implant-grade rods by the University Hospital Centre (KBC Split, Croatia), originally provided by Synthes Holding AG (Solothurn, Switzerland; West Chester, PA, USA). Cylindrical specimens (1.5 cm in length and 2.5 mm in diameter, with an exposed surface area of proximately 0.05 cm^2^) were prepared as working electrodes. Electrical contact was established by spot welding an insulated copper wire to the specimen using a micro arc welder (Lampert PUK U5, Lampert Werktechnik GmbH, Werneck, Germany), while the remaining surfaces were insulated with epoxy resin.

Sample preparation and surface treatment followed previously described procedures [7]. The surfaces were mechanically polished using SiC papers (P180 to P2000 grit), followed by final polishing with a 0.3 μm Al_2_O_3_ suspension. All materials were subjected to the same polishing protocol under identical preparation conditions to ensure comparable surface roughness and surface state prior to electrochemical testing. The samples were ultrasonically cleaned in ethanol for 5 min and rinsed with deionised water prior to electrochemical measurements. All samples were prepared immediately before testing to minimise surface contamination and uncontrolled oxide ageing.

### 2.2. Electrolyte Preparation

Corrosion tests were conducted in phosphate-buffered saline (PBS, pH 7.4) and in PBS supplemented with bovine serum albumin (BSA, 4 g/L) at 37 °C. The concentration of 4 g/L BSA was selected as a commonly used representative model albumin concentration in in vitro corrosion studies of titanium biomaterials and is consistent with concentrations used in several comparable studies investigating titanium alloys in PBS- and albumin containing media [20,22,23]. The PBS solution was prepared according to a standard composition described in the literature [7], using analytical-grade salts (NaCl, KCl, Na_2_HPO_4_, KH_2_PO_4_; Kemika, Croatia) dissolved in deionised water, with the pH adjusted to 7.4 at the test temperature. Bovine serum albumin (BSA, Fraction V, ≥96%, Sigma-Aldrich, St. Louis, MO, USA) was used as received. After BSA addition, the pH of the PBS + BSA solution remained unchanged at 7.4 ± 0.05 at 37 °C. The BSA-containing solution was freshly prepared prior to each experiment and gently stirred to ensure complete dissolution.

### 2.3. Electrochemical Measurements

Electrochemical measurements were conducted in a conventional three-electrode cell, with a saturated calomel electrode (SCE; Radiometer Analytical, France) as the reference electrode, a platinum sheet (99.9%, Sigma-Aldrich) as the counter electrode, and the sample as the working electrode. The electrolyte volume was 200 mL, and the temperature was maintained at 37 ± 0.1 °C using a thermostatically controlled circulating bath. Measurements were performed using a potentiostat/galvanostat (EG&G Princeton Applied Research Model 273A, Princeton, NJ, USA) coupled with a PAR M5210 lock-in amplifier for impedance spectroscopy measurements.

Two complementary experimental approaches were used:

- Anodic corrosion tests, performed by potentiodynamic polarisation in the range from −0.35 V vs. OCP to +2.0 V, at a scan rate of 1 mV s^−1^, after 60 min stabilisation at open circuit potential (OCP).
- Free corrosion monitoring, conducted over 9 days by recording OCP, polarisation resistance (*R*_p_), and electrochemical impedance spectroscopy (EIS). For each experiment, the same specimen remained continuously immersed in the test solution and was monitored throughout the entire 9-day period. OCP was recorded once daily prior to each *R*_p_ and EIS measurement. *R*_p_ was determined by linear polarisation within ±20 mV relative to OCP, with a scan rate of 0.2 mV s^−1^, while EIS measurements were performed in the frequency range from 50 kHz to 30 mHz using an AC amplitude of 10 mV, under automated data acquisition conditions. Impedance spectra were fitted using Boukamp’s equivalent circuit analysis program. All measurements were carried out in triplicate under naturally aerated conditions without external deaeration.

### 2.4. Surface Characterisation

After electrochemical testing, the samples were rinsed with deionised water, dried, and examined by optical microscopy (200× magnification). Surface morphology was analysed by field-emission scanning electron microscopy (FEG-SEM, Thermo Scientific Quattro ESEM, Hillsboro, OR, USA), while elemental composition was determined using energy-dispersive X-ray spectroscopy (EDS, Oxford Instruments, Abingdon, UK).

Generative AI-assisted tools (OpenAI ChatGPT, accessed in January 2026) were used solely for the assembly and layout optimization of the schematic illustration presented in Figure 14. The final figure was reviewed and refined by the authors.

## 3. Results and Discussion

The electrochemical behaviour of cp-Ti (reference material), Ti6Al7Nb and Ti6Al4V alloys was investigated in PBS solution at 37 °C, with albumin as a model protein. The results provide insight into the stability of oxide films and their interaction with proteins under biologically relevant conditions.

### 3.1. Anodic Behaviour of Ti6Al7Nb and Ti6Al4V in Albumin-Containing PBS

**Figure 1:**
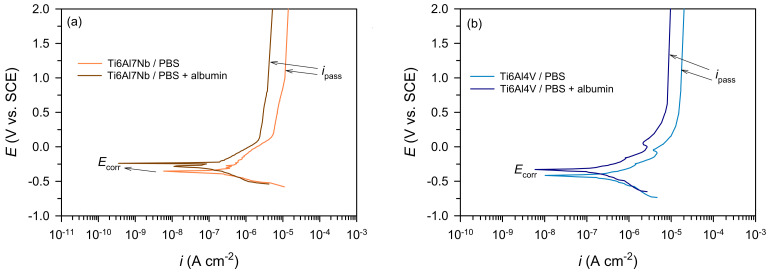
Potentiodynamic polarisation curves of the tested samples after 1 h exposure to PBS and PBS + albumin solutions for: (**a**) Ti6Al7Nb (**b**) Ti6Al4V (T = 37 °C).

The anodic behaviour of Ti6Al7Nb and Ti6Al4V was evaluated using the potentiodynamic method (–0.35 V vs. OCP to 2.0 V) after 60 min stabilisation at OCP (Figure 1). The polarisation curves show a clear influence of albumin on the passivation behaviour of both alloys compared to pure PBS.

The corrosion potential (*E*_corr_) provides qualitative information on the thermodynamic tendency for corrosion; however, it cannot be used as a direct measure of corrosion resistance, particularly for passive systems such as titanium alloys. Both alloys exhibit typical passive behaviour of titanium, characterised by a transition from active dissolution to a stable passive region with nearly constant current density [6,7,8,9]. The absence of transpassive breakdown up to ≈2.0 V indicates high anodic stability of the formed oxide films.

*E*_corr_, *i*_corr_, and *i*_pass_ were determined from the PD curves (Table 1). The corrosion current density (*i*_corr_) was determined by Tafel extrapolation of the linear anodic and cathodic branches in the near *E*_corr_ region prior to passivation, while *i*_pass_ was taken from the anodic polarization curve at a fixed potential within the stable passive region (approximately 1.0–1.2 V vs. SCE), where the current density remained nearly constant. The results show that Ti6Al7Nb exhibits lower *i*_corr_ and *i*_pass_ values and a more positive *E*_corr_ compared to Ti6Al4V, indicating a more stable and protective passive film and higher corrosion resistance, which is consistent with literature reports on the beneficial effect of Nb addition in titanium alloys [28,29].

The addition of albumin shifts *E*_corr_ to more positive values and reduces *i*_corr_ and *i*_pass_, indicating a predominantly inhibitory effect due to protein adsorption [13,17]. This suggests the formation of a more compact and protective surface layer that limits charge transfer and ionic transport. The effect of albumin is particularly pronounced for Ti6Al4V, where a significant decrease in *i*_corr_ and *i*_pass_ is observed, indicating that protein adsorption has a stronger relative impact on this alloy. This behaviour can be attributed to the microstructural heterogeneity of Ti6Al4V (α + β phases), which provides preferential sites for protein adsorption and subsequent local stabilisation [13,14]. Thus, the observed improvement does not necessarily imply intrinsically superior corrosion resistance, but rather reflects a greater sensitivity of this alloy to adsorbed proteins.

The PD results confirm the positive influence of albumin on the corrosion resistance of both tested materials, while also indicating that albumin partially reduces the differences in corrosion behaviour between the two alloys, without completely eliminating them.

#### Surface Analysis After PD Measurements

**Figure 2:**
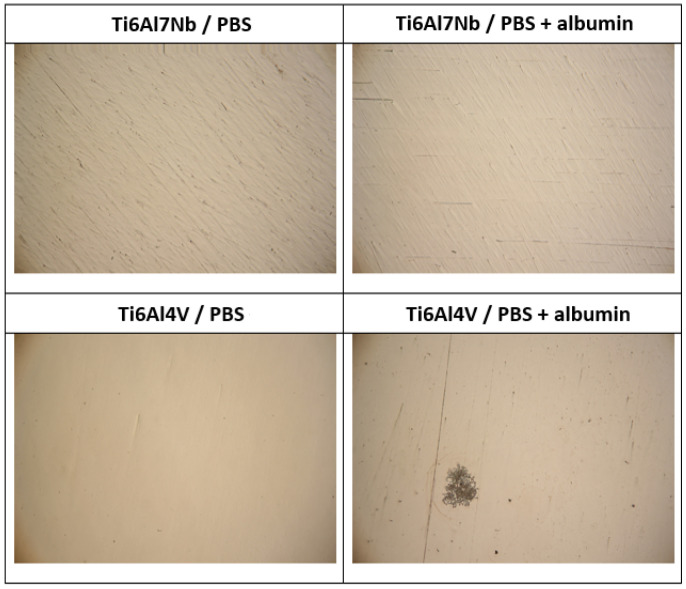
Optical microscopy images of Ti6Al7Nb and Ti6Al4V surfaces after potentiodynamic polarisation in PBS and PBS + albumin solution at 37 °C.

Optical microscopy was used (Figure 2) to gain a general understanding of the surface condition after potentiodynamic polarisation. The surfaces remained compact, without visible macroscopic damage or signs of localised corrosion, even after exposure to high anodic potentials (≈2 V), which significantly exceed physiological conditions. This behaviour suggests a high degree of stability and preservation of the passive oxide layer over a wide anodic potential range [6,7,8,9].

Detailed characterisation of the surface was performed by SEM/EDS analysis (Table 2 and Table 3), which confirms the stability of the passive films and provides insight into the effect of albumin adsorption on surface composition.

After polarisation in PBS solution, Ti and O are predominant in both alloys, with smaller amounts of Na, Cl, P, and K, indicating a preserved passive oxide layer. The relatively high proportion of titanium (≈75–72%) indicates that no significant breakdown of the surface film occurred even at high anodic potentials. Alloying elements (Al, Nb, or V) are present in both alloys, confirming the multicomponent nature of the oxide layer, consistent with the literature on the complex nature of oxide films on titanium alloys [10,28].

In systems containing albumin, the relative proportion of metal and oxide signals decreases, while the proportion of C, N, and S increases, indicating the presence of an adsorbed protein-containing layer on the surface [13,17]. A decrease in Ti signal intensity further suggests partial surface coverage by an organic layer.

In Ti6Al7Nb, this effect is evident as an increase in carbon content (≈13–20%) and nitrogen (≈0.8–1.9%), with a simultaneous decrease in titanium content (≈53–64%), indicating relatively uniform adsorption of the protein-containing layer. SEM images show a more pronounced fine surface relief, while the substrate microstructure remains partially visible, suggesting a thin but continuous organic layer. This distribution is consistent with the formation of a relatively uniform (but not fully homogeneous) adsorbed layer covering the oxide surface, in agreement with literature reports [13,16].

Albumin adsorption was also confirmed in Ti6Al4V, with C (≈8–16%) and N (≈0.5–1.1%). However, a larger range of titanium content (≈52–70%) suggests non-uniform surface coverage, i.e., the presence of areas with a thinner or discontinuous protein-containing layer. SEM images indicate locally more uniform regions, but combined with EDS results this points to variable thickness and distribution of the organic layer. Such heterogeneity is often associated with chemical and microstructural non-uniformity of the oxide layer, especially in multicomponent alloys such as Ti6Al4V [10,13,28]. This observation is consistent with electrochemical results, which showed a more pronounced relative effect of albumin on Ti6Al4V, likely due to preferential adsorption at more reactive or heterogeneous surface sites.

Although potentiodynamic measurements indicate high stability of the passive layer under extreme anodic conditions, these conditions do not fully represent physiological environments. Therefore, long-term exposure in protein-containing solutions was investigated. Accordingly, time-dependent monitoring of spontaneous corrosion and surface analysis after prolonged exposure were performed.

### 3.2. Free Corrosion of Ti6Al4V and Ti6Al7Nb in Albumin-Containing PBS

Free corrosion of cp-Ti, Ti6Al7Nb, and Ti6Al4V alloys was investigated in PBS solution, with and without albumin, at 37 °C over nine days. Open-circuit potential, polarization resistance, and impedance measurements were used to monitor the evolution of oxide film stability and protein–surface interactions relevant to long-term implant performance [12,13,14]. Together, these complementary electrochemical methods provide a consistent basis for comparing corrosion behaviour among the investigated materials.

#### 3.2.1. Change in Open-Circuit Potential

**Figure 3:**
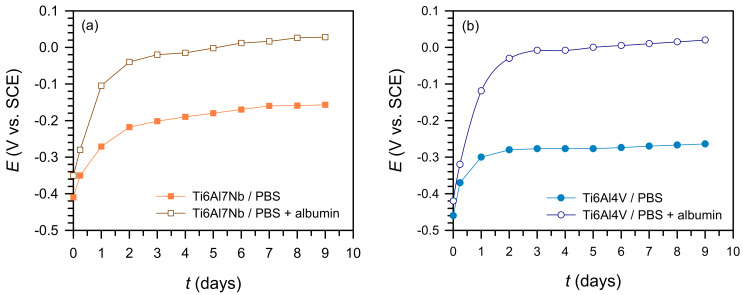
Change in open circuit potential of the tested samples in PBS solution and PBS + albumin solution for: (**a**) Ti6Al7Nb and (**b**) Ti6Al4V.

As shown in Figure 3, both materials exhibit an increase in OCP towards more positive values immediately after immersion, consistent with the rapid establishment and stabilization of the passive oxide film [6,7,8,9]. After this initial phase, the OCP stabilises, reflecting a dynamic balance between oxide formation and dissolution [10,11]. In PBS media, phosphate ions may also contribute to passive film stabilisation by adsorbing onto titanium oxide surfaces, thereby influencing both oxide growth and subsequent protein adsorption.

The final OCP values are: −0.161 V (Ti6Al7Nb/PBS), +0.028 V (Ti6Al7Nb/PBS + albumin), −0.270 V (Ti6Al4V/PBS), and −0.090 V (Ti6Al4V/PBS + albumin).

The positive shift of OCP in the presence of albumin suggests modification of the oxide/electrolyte interface due to protein adsorption, consistent with reduced surface reactivity under the investigated conditions [13,14]. Corrosion resistance was evaluated based on kinetic parameters (*R*_p_, *i*_corr_, and EIS results), rather than OCP values alone. These positive OCP shifts are consistent with the simultaneous increase in *R*_p_ and *R*_2_ values, confirming that all electrochemical techniques indicate the same corrosion resistance trend.

#### 3.2.2. Linear Polarisation and Change in *R*_p_ Value

**Figure 4:**
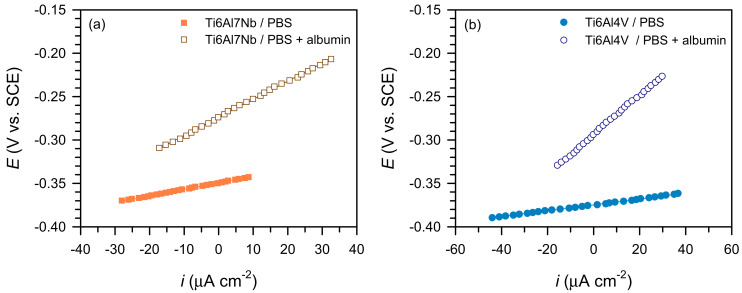
Linear polarisation curves of the tested samples after 1 h exposure to PBS and PBS + albumin solutions for: (**a**) Ti6Al7Nb and (**b**) Ti6Al4V (T = 37 °C).

The linear polarisation method (±20 mV vs. OCP) enabled monitoring of corrosion behaviour over time. The results (Figure 4) show a linear *i*–*E* relationship, where the slope (Δ*E*/Δ*i*) represents *R*_p_, reflecting corrosion resistance.

**Figure 5:**
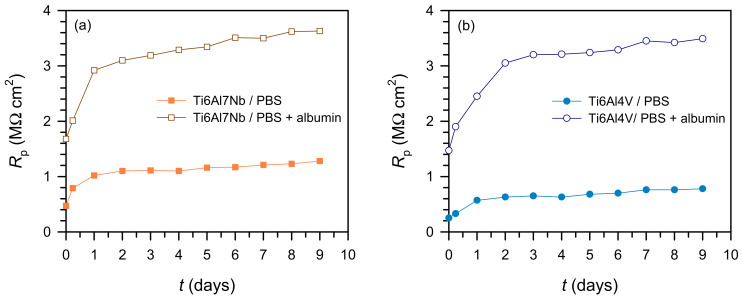
Time dependence of *R*_p_ of the tested samples in PBS solution and PBS + albumin solution for: (**a**) Ti6Al7Nb and (**b**) Ti6Al4V.

Figure 5 shows the change in *R*_p_ over time. All systems follow a similar trend—a sharp increase during the first two days, followed by stabilisation. The addition of albumin increases *R*_p_ for both materials, confirming enhanced surface stability due to protein adsorption. This behaviour is consistent with rapid initial changes in passive film properties, followed by establishment of a quasi-steady electrochemical state.

The *R*_p_ values increase in the following order: Ti6Al4V/PBS < Ti6Al4V/PBS + albumin < Ti6Al7Nb/PBS < Ti6Al7Nb/PBS + albumin < cp-Ti/PBS < cp-Ti/PBS + albumin.

cp-Ti shows the highest corrosion resistance; however, in the presence of albumin, the difference between Ti6Al4V and cp-Ti is significantly reduced, indicating a strong stabilising effect of protein adsorption on the more heterogeneous alloy surface. Thus, albumin mitigates intrinsic microstructural differences without completely eliminating them.

Key electrochemical and impedance parameters after prolonged exposure (9 days) are summarised in Table 4.

Albumin enhances corrosion resistance in both alloys, with a more pronounced effect in Ti6Al4V, reflecting its greater sensitivity to surface modification by adsorbed proteins [17,21]. Since albumin was introduced into phosphate-containing PBS rather than a phosphate-free electrolyte, the observed improvements reflect the combined influence of phosphate ions and protein adsorption, and their individual contributions cannot be fully distinguished under the present experimental design.

#### 3.2.3. Electrochemical Impedance Spectroscopy

**Figure 6:**
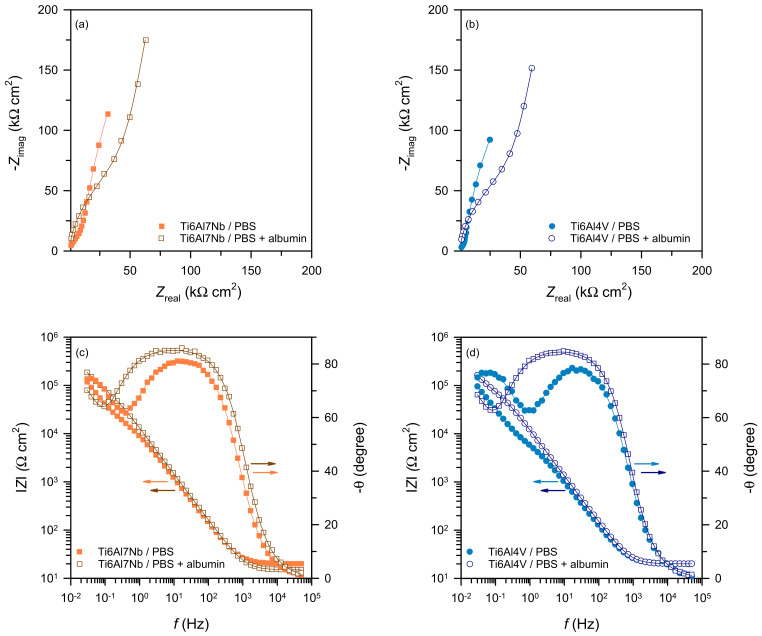
Nyquist (**a**,**b**) and Bode (**c**,**d**) plots recorded on a Ti6Al7Nb and Ti6Al4V alloys in PBS and PBS + albumin solutions 9 days at OCP (T = 37 °C).

Figure 6 shows the Nyquist Figure 6a,b and Bode Figure 6c,d plots of Ti6Al7Nb and Ti6Al4V alloys recorded after 9 days of exposure in PBS and PBS + albumin solutions at 37 °C.

The Nyquist plots exhibit a smaller capacitive semicircle at high frequencies and a larger, incomplete semicircle at low frequencies, while the Bode plots show two phase maxima. The presence of two time constants suggests two electrochemical processes, commonly associated with a bilayer oxide structure on titanium surfaces, consisting of a porous outer layer and a compact inner barrier layer [30,31,32,33].

The high impedance values (on the order of 10^6^ Ω·cm^2^ at low frequencies) and well-defined phase maxima confirm the formation of a stable and protective oxide layer under the investigated conditions. Ti6Al7Nb exhibits higher impedance values than Ti6Al4V, reflecting better protective properties of its oxide film. This trend is in full agreement with the preceding OCP and *R*_p_ observations, further confirming the superior electrochemical stability of Ti6Al7Nb across all applied techniques.

**Figure 7:**
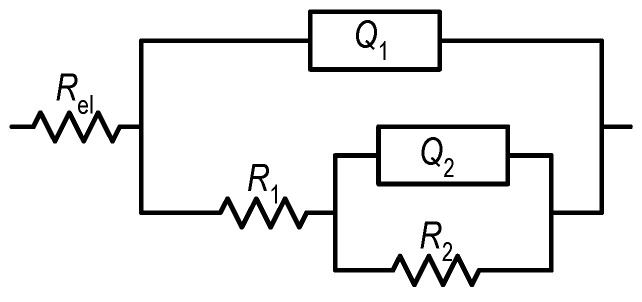
Applied equivalent circuit.

Deviation from ideal capacitive behaviour is observed, as the parameter *n* is lower than unity. Therefore, the interfacial response is described using a constant phase element (CPE, *Q*), reflecting surface inhomogeneity and non-ideal dielectric properties of the oxide film [34]. The spectra were fitted using the equivalent electrical circuit shown in Figure 7 [30,31,32,33], consisting of the electrolyte resistance (*R*_el_ ≈ 20 Ω·cm^2^) in series with two time constants (*Q*_1_*R*_1_ and *Q*_2_*R*_2_), corresponding to the porous outer layer and the compact barrier layer, respectively. This equivalent circuit was selected because it is commonly accepted for passive titanium systems exhibiting two electrochemical time constants [30,31,32,33], and it provided the best agreement with the experimental impedance response in all investigated cases. The impedance spectra were analysed using Boukamp’s EIS fitting software (version 3.97), and the quality of the simulations was confirmed by low χ^2^ values in the range of 10^−3^–10^−2^, indicating satisfactory agreement between experimental and simulated data.

**Figure 8:**
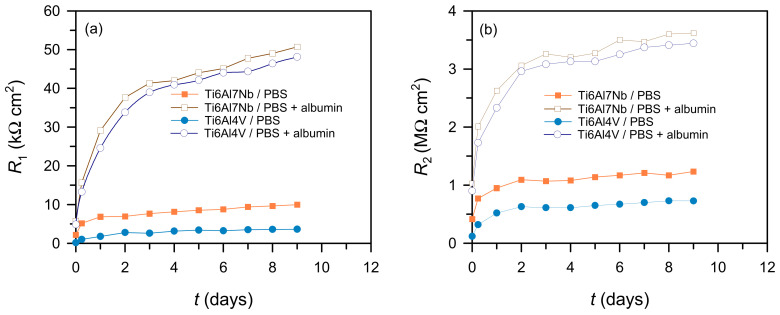
Dependence of the resistance of the porous (**a**) and barrier layer (**b**) on Ti6Al7Nb and Ti6Al4V over time in the tested solutions.

**Figure 9:**
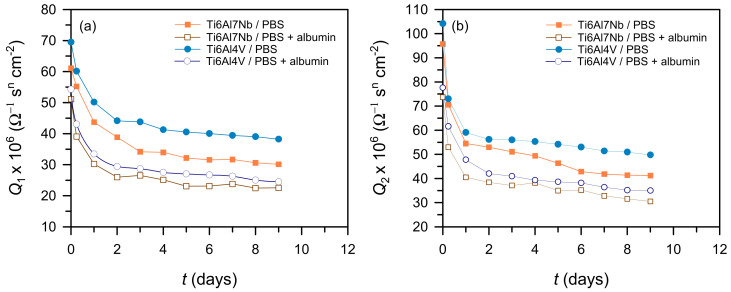
Dependence of the capacitance of the porous (**a**) and barrier layer (**b**) on Ti6Al7Nb and Ti6Al4V over time in the tested solutions.

The fitted parameters (Figure 8 and Figure 9) show that the barrier layer provides the dominant contribution to corrosion protection, as *R*_2_ values are significantly higher than *R*_1_. A good agreement was also observed between the polarisation resistance values obtained from linear polarization measurements and the sum of the fitted impedance resistances (*R*_1_ + *R*_2_), both of which were of similar magnitude across all investigated systems. This consistency confirms the reliability of the equivalent circuit model and demonstrates that both electrochemical methods yield the same trends in corrosion resistance.

In Ti6Al7Nb, an increase in *R*_2_ and a decrease in *Q*_2_ with time indicate progressive stabilisation and densification of the oxide film, while the increase in *R*_1_ suggests reduced electrolyte penetration into the porous outer layer. Ti6Al4V shows similar trends but with lower resistance and higher *Q*, indicating a less compact, more heterogeneous oxide film, resulting in lower protective performance. In contrast, cp-Ti exhibits the highest resistance and lowest *Q* values, consistent with the formation of a more stable oxide layer.

The influence of albumin is evident from the fitted parameters rather than directly from the spectra. In its presence, both alloys show an increase in *R*_1_ and *R*_2_ and a decrease in *Q*_1_ and *Q*_2_, indicating improved surface coverage and reduced ion transport. This effect is more pronounced for Ti6Al4V, suggesting preferential interaction with more heterogeneous surfaces [13,14,26]. As a result, the differences in resistance values between the alloys decrease in the presence of albumin, although Ti6Al7Nb retains higher absolute resistance.

It should be noted that these electrochemical parameters provide indirect evidence of interfacial modification and do not allow direct determination of the spatial uniformity of the adsorbed protein layer.

**Figure 10:**
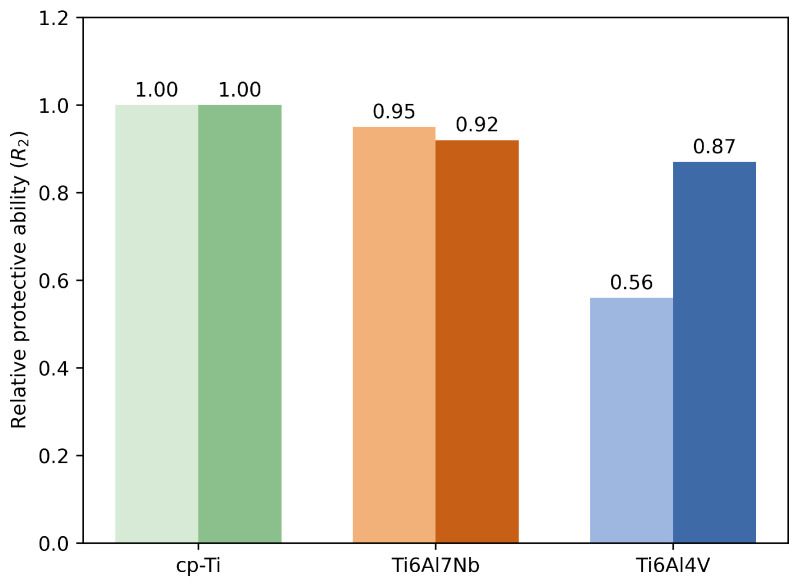
Relative protective ability (*R*_2_) of cp-Ti, Ti6Al7Nb, and Ti6Al4V after 9 days of immersion in PBS and PBS with albumin. Values are normalized to cp-Ti in each medium and show comparative ratios based on the fitted mean *R*_2_. Albumin causes minor changes for Ti6Al7Nb and a marked increase for Ti6Al4V, highlighting alloy-dependent responses to protein adsorption.

The relative protective ability shown in Figure 10 was evaluated using the *R*_2_ parameter obtained after 9 days of immersion. The values were normalized to commercially pure titanium (cp-Ti), which was taken as the reference material (*R*_2_ = 1) for each corresponding medium.

As shown in Figure 10, albumin affects the alloys differently. While cp-Ti exhibits nearly unchanged protective ability in both media, Ti6Al7Nb shows only a slight decrease in normalized *R*_2_, indicating relatively stable behaviour with low sensitivity to protein adsorption. However, Ti6Al4V exhibits a pronounced increase in normalized *R*_2_ in the presence of albumin, reflecting greater sensitivity to protein adsorption and a more pronounced modification of its surface protective properties.

These results indicate that Ti6Al7Nb maintains more stable protective behaviour under changing environmental conditions, whereas Ti6Al4V shows a marked increase in protective ability in response to albumin, demonstrating a dynamic and surface-sensitive adjustment.

#### 3.2.4. SEM/EDS Surface Analysis After Free Corrosion

**Figure 11:**
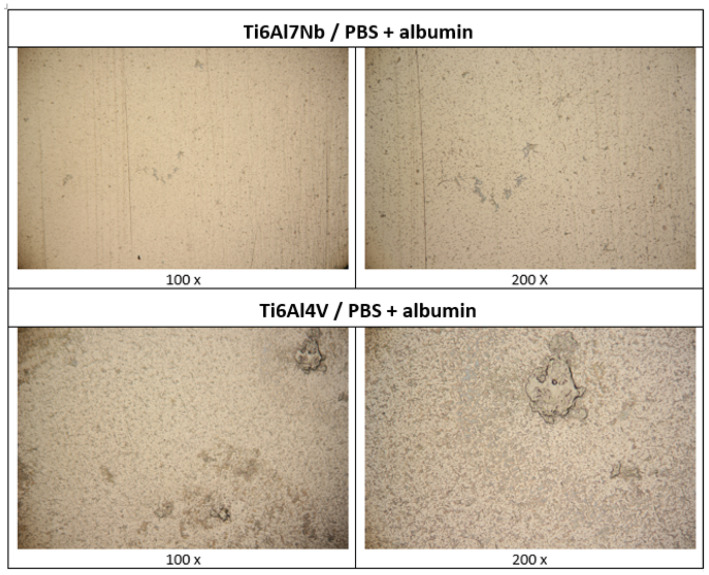
Optical microscopic images of the surfaces of the tested samples after 9 days of exposure to PBS and PBS + albumin solution at 37 °C.

Optical microscopy was used to gain a general understanding of the surface appearance after nine days of exposure to the PBS + albumin solution (Figure 11). On Ti6Al7Nb, a relatively uniform surface was observed without pronounced local clusters, indicating a more uniform distribution of the adsorbed layer. In contrast, Ti6Al4V exhibited more pronounced surface heterogeneity, with visible local clusters and an uneven distribution of surface features, consistent with localised protein adsorption. Due to limited resolution, optical microscopy was used only for qualitative assessment.

**Figure 12:**
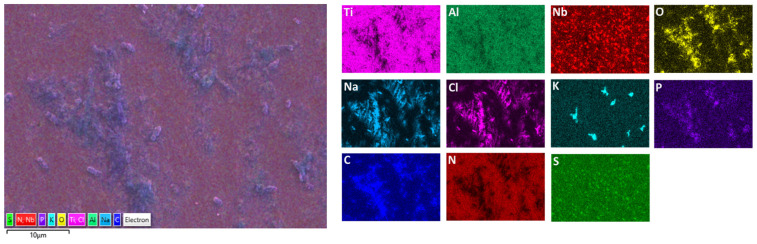
EDS surface mapping of Ti6Al7Nb after exposure to PBS + albumin solution.

**Figure 13:**
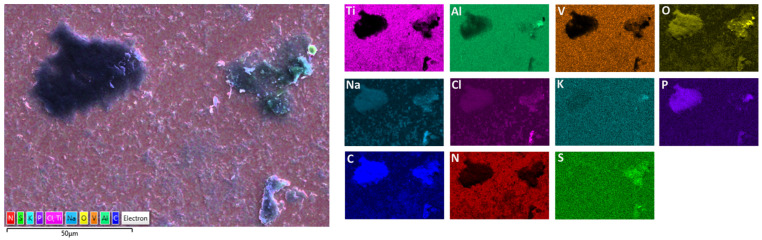
EDS surface mapping of Ti6Al4V after exposure to PBS + albumin solution.

A detailed surface analysis was performed using SEM/EDS (Table 5) and elemental mapping (Figure 12 and Figure 13), providing insight into surface composition, element distribution, and protein adsorption behaviour.

A relatively uniform surface composition was observed for Ti6Al7Nb. The titanium content ranges from approximately 41 to 57 wt.%, while carbon varies between ~15 and 30 wt.% across all analysed points, without pronounced local enrichment. The oxygen content remains relatively stable (≈12–20 wt.%), indicating a continuous oxide layer covered by a relatively uniform protein film. Nitrogen and sulfur are present in low amounts, confirming the presence of adsorbed protein species [18,35].

In contrast, Ti6Al4V exhibits pronounced spatial variability. Carbon content shows significant fluctuations, reaching up to ~50 wt.% in certain regions, while in other areas it decreases below 10 wt.%. These variations are accompanied by large changes in titanium content (from ~17 to ~80 wt.%) and oxygen (≈5–20 wt.%), indicating alternating regions of thicker protein accumulation and areas with thinner adsorbed protein coverage, where the oxide surface signal remains more pronounced. Regions with high carbon content correspond to darker areas observed in SEM images, indicating locally thicker protein layers, which is further confirmed by elemental mapping showing carbon-rich “hot spots” [18,35]. This behaviour confirms non-uniform protein adsorption associated with surface heterogeneity [28,29,35].

Electrolyte elements (Na, Cl, P, K) are present in low amounts in both alloys; however, Ti6Al4V shows higher variability and locally increased concentrations, suggesting retention of electrolyte species within heterogeneous surface regions [18,29].

Overall, the results indicate a more uniform (but not fully homogeneous) protein layer on Ti6Al7Nb, while Ti6Al4V exhibits non-uniform adsorption characterized by a generally adsorbed surface layer with localized regions of enhanced protein accumulation, consistent with its more heterogeneous oxide surface [18,28,29,35]. The observed differences are further influenced by the specific alloying elements present in the investigated materials. In Ti6Al7Nb, niobium contributes to the formation of stable Nb_2_O_5_ within the passive film, which enhances oxide stability and promotes a more uniform surface structure. This behaviour is consistent with the generally higher thermodynamic stability of niobium-containing oxide species, which favours formation of a more stable passive film. This contributes to the more uniform protein adsorption and electrochemical response observed for this alloy. In contrast, Ti6Al4V contains vanadium, whose oxide species are generally less stable under physiological conditions and may contribute to increased surface heterogeneity. As a result, localized variations in oxide composition and surface charge promote non-uniform protein adsorption, leading to more heterogeneous corrosion-related behaviour. Aluminium contributes to oxide formation in both alloys, but the contrasting effects of niobium and vanadium are considered the dominant factors governing the observed differences.

Elemental mapping provides insight into the spatial distribution of elements and complements the point EDS analysis (Figure 12 and Figure 13).

On Ti6Al7Nb (Figure 12), a diffuse and relatively uniform distribution of carbon, nitrogen, and electrolyte elements (Na, Cl, P) is observed, without pronounced clustering, in agreement with the uniform composition observed in point analysis and indicating relatively uniform protein adsorption [18,35]. In contrast, Ti6Al4V (Figure 13) shows pronounced microscale heterogeneity, with localised areas of increased carbon content (“hot spots”), indicating non-uniform protein distribution. However, weaker carbon and nitrogen signals are also detected across the surrounding surface, suggesting that protein adsorption occurs over the entire surface, with localized regions of enhanced accumulation. Electrolyte elements (Na, Cl, P) also show localised enrichment, often associated with these regions, suggesting their retention within thicker protein layers or surface irregularities.

A more detailed analysis shows that nitrogen does not fully coincide spatially with carbon but is more pronounced in areas where the metal signal is still present, suggesting overlapping contributions from the adsorbed protein layer and the underlying substrate. Sulfur appears in small, discrete regions, typically associated with higher carbon content, supporting the presence of adsorbed proteins [18,35]. Since mapping was not performed at identical magnifications, the comparison is limited to qualitative assessment of spatial distribution rather than feature size.

The observed differences are consistent with variations in oxide layer composition and surface heterogeneity. Differences in isoelectric point (IEP) of constituent oxides (e.g., TiO_2_ ≈ 5–6, Al_2_O_3_ ≈ 8–9, V_2_O_5_ ≈ 2–3) may influence local surface charge and thereby affect protein adsorption behaviour [36,37,38]. However, this effect cannot be directly confirmed by SEM/EDS and is therefore only suggested.

The SEM/EDS mapping results suggest that Ti6Al7Nb exhibits a more uniform surface and more uniformly distributed protein layer, whereas Ti6Al4V shows greater microstructural non-uniformity and localised protein accumulation. These findings are consistent with the electrochemical results and further highlight the role of surface characteristics in corrosion behaviour under biological conditions [18,28,29,35,37].

The results also confirm that albumin exerts a predominantly inhibitory effect on corrosion under the investigated conditions, as reflected by increased impedance, decreased corrosion current density, and a positive shift of the corrosion potential. This behaviour is consistent with literature showing that proteins can stabilise the passive oxide layer and act as a diffusion barrier under static conditions [13,17,35].

These alloy-dependent differences further emphasise the importance of oxide composition and structure: Ti6Al7Nb exhibits a more stable electrochemical response, while Ti6Al4V shows less uniform interfacial behaviour associated with spatially uneven adsorption. Compared with cp-Ti, both alloys show lower corrosion resistance; however, the difference between cp-Ti and Ti6Al7Nb decreases in the presence of albumin, indicating additional stabilisation by the adsorbed protein layer.

Overall, the results confirm that under static, biologically relevant conditions, proteins enhance the protective properties of the oxide layer, with the final effect governed by the interplay between oxide stability, surface heterogeneity, and protein–surface interactions [13,17,21].

**Figure 14:**
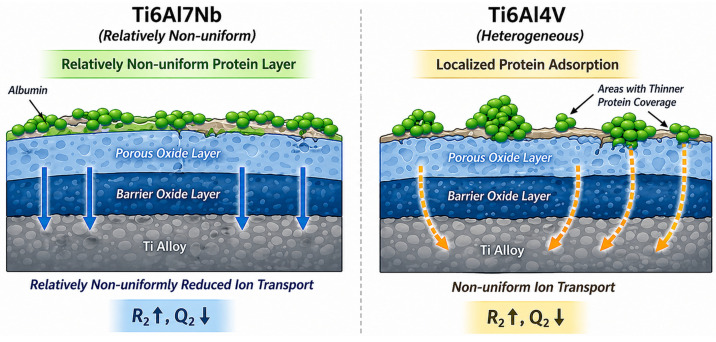
Schematic representation of the oxide–protein interface on Ti6Al7Nb and Ti6Al4V alloys in albumin-containing PBS at 37 °C, based on electrochemical impedance analysis, equivalent circuit modelling, and SEM/EDS observations. The barrier and porous oxide layers are inferred from the two-time-constant EIS response and are not directly observed experimentally. Ti6Al7Nb shows a comparatively more uniform (but not perfectly homogeneous) protein distribution, whereas Ti6Al4V exhibits non-uniform adsorption with localized regions of enhanced accumulation, consistent with SEM/EDS trends. The improved protective behaviour is associated with increased charge transfer resistance and decreased capacitance of the barrier layer (*R*_2_ ↑, *Q*_2_ ↓).

The results can be interpreted within a unified physical model of the metal–electrolyte interface, schematically illustrated in Figure 14. Under the investigated conditions, the surface is interpreted, based on EIS analysis and equivalent circuit modelling, as comprising an inner barrier oxide layer and an outer porous oxide layer, in accordance with commonly accepted electrochemical models for passive titanium systems [30,31,32,33].

The barrier layer governs charge transfer resistance, while the porous outer layer controls electrolyte access to the surface. Protein adsorption is interpreted as forming an interfacial layer that may partially block transport pathways and modify the dielectric properties of the interface, contributing to increased resistance and decreased capacitance in EIS measurements (*R*_2_ ↑, *Q*_2_ ↓).

The extent and spatial distribution of this effect are strongly governed by surface heterogeneity. As illustrated in Figure 14, Ti6Al7Nb is associated with a comparatively more uniform (but not fully homogeneous) protein distribution, resulting in more evenly distributed surface coverage and more consistently reduced ion transport. In contrast, Ti6Al4V shows greater surface heterogeneity that promotes more heterogeneous adsorption patterns, leading to protein-rich regions interspersed with areas of thinner coverage. This results in non-uniform ion transport pathways and spatially variable protective behaviour. It should be noted that the term “uniform” refers to a comparatively more even spatial distribution rather than a perfectly continuous homogeneous layer.

The agreement between electrochemical parameters (increase in *R*_2_, decrease in *Q*_2_), surface composition (variations in C, Ti, and O), and spatial distribution of elements provides a consistent interpretation of the system. Overall, protein adsorption acts as a stabilising interfacial factor that reduces ion transport and enhances corrosion resistance, while surface heterogeneity governs the spatial distribution and effectiveness of this protection.

## 4. Conclusions

The electrochemical and surface behaviour of cp-Ti, Ti6Al7Nb, and Ti6Al4V alloys was systematically compared in PBS and PBS containing albumin under static in vitro conditions at 37 °C. Under all investigated conditions, the corrosion resistance followed the order Ti6Al4V < Ti6Al7Nb < cp-Ti, indicating higher stability of cp-Ti and Ti6Al7Nb compared to Ti6Al4V.

The presence of albumin exerted a predominantly beneficial effect on all tested materials, reflected in lower corrosion current densities, higher polarisation resistance, and increased impedance-derived resistance values. This effect was most pronounced for Ti6Al4V, where the barrier-layer resistance *R*_2_ increased from 0.73 to 3.44 MΩ·cm^2^ in the presence of albumin, compared with an increase from 1.24 to 3.62 MΩ·cm^2^ for Ti6Al7Nb, indicating that Ti6Al4V is more sensitive to protein adsorption and surface modification than Ti6Al7Nb.

A notable finding of this study is the alloy-dependent effect of albumin on passive film stabilisation. Although Ti6Al4V remained the least corrosion-resistant alloy overall, it exhibited the most pronounced improvement in electrochemical parameters in the presence of albumin, indicating greater sensitivity to protein-mediated surface modification. In contrast, Ti6Al7Nb showed smaller changes, consistent with a more stable and less environmentally sensitive passive film. This difference highlights distinct adsorption-driven responses of the investigated alloys.

Surface analysis showed that Ti6Al7Nb promoted a relatively uniform (but not fully homogeneous) protein distribution, reflected by more uniform carbon and nitrogen surface signals, whereas Ti6Al4V exhibited non-uniform adsorption characterised by localised carbon-rich regions indicating enhanced protein accumulation. These differences are consistent with the observed electrochemical behaviour and highlight the role of surface heterogeneity in governing protein adsorption and oxide film stability.

The results further indicate that protein adsorption acts as a stabilizing interfacial factor under the investigated conditions, reducing ion transport across the oxide/electrolyte interface and improving the protective properties of passive films. However, these findings are limited to simplified static in vitro conditions and should not be directly extrapolated to dynamic physiological environments involving fluid flow, tribocorrosion, cyclic loading, or inflammatory species.

Overall, the study demonstrates that Ti6Al7Nb provides a more stable and predictable electrochemical response in albumin-containing PBS than Ti6Al4V, suggesting its potential as a more stable alternative under the investigated static in vitro conditions.

## Figures and Tables

**Table 1:** Corrosion parameters for cp-Ti, Ti6Al7Nb, and Ti6Al4V alloy in PBS solution, without and in the presence of albumin.

Sample	*E*_corr_ (V)	*i*_corr_ (µA·cm^−2^)	*i*_pass_ (µA·cm^−2^)
cp-Ti/PBS	−0.31	0.19	9.46
cp-Ti/PBS + albumin	−0.17	0.12	3.56
Ti6Al7Nb/PBS	−0.37	0.20	10.08
Ti6Al7Nb/PBS + albumin	−0.22	0.16	4.05
Ti6Al4V/PBS	−0.43	0.31	18.16
Ti6Al4V/PBS + albumin	−0.34	0.19	7.22

**Table 2:** SEM images and semi-quantitative elemental composition (at marked positions) of Ti6Al7Nb and Ti6Al4V surfaces after PD measurements in PBS solution at 37 °C.

	Spectrum				
Ti6Al7Nb/PBS	Element (wt.%)	1	2	3	4
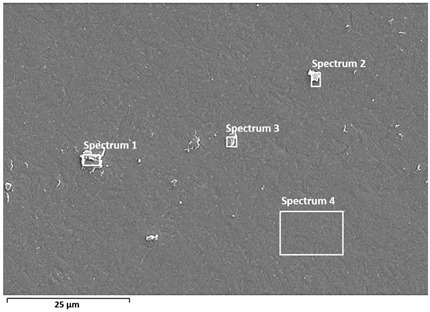	C	1.80	2.02	1.08	1.56
	O	7.54	7.53	9.80	6.35
	Na	1.21	0.99	0.75	0.21
	P	4.20	3.51	3.89	3.34
	Cl	5.56	5.08	4.89	6.04
	K	0.37	0.58	0.43	1.11
	Ti	71.57	72.74	71.45	71.96
	Al	3.79	3.50	3.72	5.07
	Nb	3.96	4.05	4.02	4.36
	Total	100.00	100.00	100.00	100.00
**Ti6Al4V/PBS**	**Spectrum**				
	**Element (wt.%)**	**1**	**2**	**3**	**4**
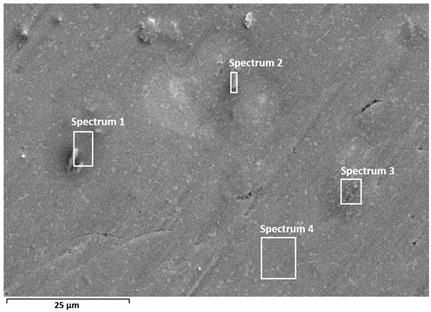	C	1.85	1.97	1.38	1.50
	O	17.61	8.64	15.17	9.39
	Na	1.33	0.26	0.44	1.07
	P	1.94	6.03	1.41	4.02
	Cl	3.58	5.35	3.72	4.34
	K	0.32	0.54	0.23	0.61
	Ti	64.92	70.97	70.24	72.01
	Al	5.59	2.92	4.48	4.69
	V	2.86	3.32	2.93	2.37
	Total	100.00	100.00	100.00	100.00

**Table 3:** SEM images and semi-quantitative elemental composition (at marked positions) of Ti6Al7Nb and Ti6Al4V surfaces after PD measurements in PBS + albumin solution at 37 °C.

	Spectrum				
Ti6Al7Nb/PBS + Albumin	Element (wt.%)	1	2	3	4
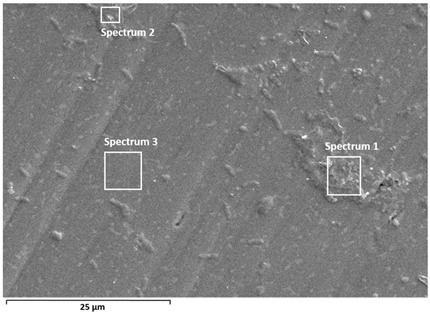	C	20.44	18.19	13.69	
	N	1.88	1.14	0.79	
	O	13.11	11.21	8.02	
	Na	0.20	0.11	0.05	
	P	0.93	1.67	2.04	
	S	0.26	0.31	0.13	
	Cl	2.01	2.42	3.18	
	K	0.06	0.13	0.15	
	Ti	53.47	57.39	63.56	
	Al	4.87	4.29	5.16	
	Nb	2.77	3.14	3.23	
	Total	100.00	100.00	100.00	
	**Spectrum**				
**Ti6Al4V/PBS + albumin**	**Element (wt.%)**	**1**	**2**	**3**	**4**
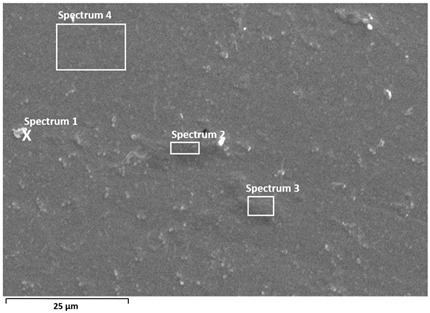	C	15.31	16.12	14.08	7.89
	N	0.76	1.06	0.89	0.53
	O	19.32	14.73	9.21	8.33
	Na	0.49	0.11	0.21	0.77
	P	2.08	3.01	1.07	2.94
	S	0.17	0.20	0.17	0.11
	Cl	2.61	1.64	3.72	2.22
	K	0.32	0.23	0.26	0.34
	Ti	52.21	56.18	63.05	69.30
	Al	4.55	3.92	4.48	5.36
	V	2.18	3.01	2.77	3.25
	Total	100.00	100.00	100.00	100.00

**Table 4:** Summary of electrochemical and impedance parameters (OCP, *R*_p_, *R*_1_, *R*_2_, *Q*_1_ and *Q*_2_) of Ti6Al7Nb, Ti6Al4V and cp-Ti after 9 days of exposure in PBS and PBS + albumin solutions at 37 °C.

Sample	OCP (V)	*R*_p_ (MΩ·cm^2^)	*Q*_1_ × 10^6^ (Ω^−1^·s^n^·cm^−2^)	*n*_1_	*R*_1_ (kΩ·cm^2^)	*Q*_2_ × 10^6^ (Ω^−1^·s^n^·cm^−2^)	*n*_2_	*R*_2_ (MΩ·cm^2^)
cp-Ti/PBS	−0.11	1.51	30.09	0.94	10.44	41.76	0.95	1.31
cp-Ti/PBS + albumin	0.114	4.22	20.87	0.96	55.32	28.64	0.98	3.95
Ti6Al7Nb/PBS	−0.16	1.37	30.15	0.93	9.97	41.24	0.94	1.24
Ti6Al7Nb/PBS + albumin	0.028	3.76	22.53	0.95	50.71	30.49	0.97	3.62
Ti6Al4V/PBS	−0.27	0.79	38.23	0.91	3.64	49.77	0.94	0.73
Ti6Al4V/PBS + albumin	−0.09	3.47	24.49	0.95	48.10	34.98	0.96	3.44

**Table 5:** SEM images and semi-quantitative elemental composition (at marked positions) of Ti6Al4V and Ti6Al7Nb surfaces after prolonged exposure (9 days) to PBS + albumin solution at 37 °C.

	Spectrum						
Ti6Al7Nb/PBS + Albumin	Element (wt.%)	1	2	3	4	5	6
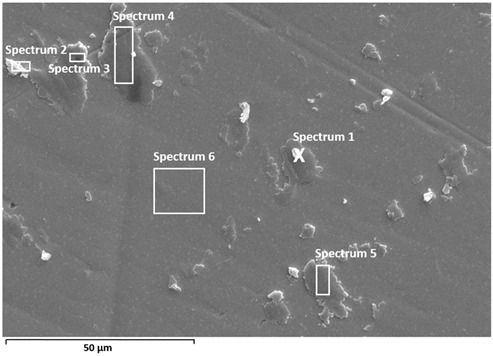	C	29.93	27.12	20.65	22.76	18.33	15.18
	N	1.23	1.35	0.95	1.01	1.09	0.74
	O	20.21	19.45	17.43	14.33	12.66	13.55
	Na	0.22	0.12	0.24	0.31	0.54	0.61
	P	1.09	1.01	1.72	1.54	2.05	2.43
	S	0.27	0.25	0.20	0.14	0.29	0.10
	Cl	1.11	1.24	1.65	1.48	1.89	1.95
	K	0.21	0.20	0.19	0.19	0.18	0.22
	Ti	41.22	43.86	50.96	52.34	54.73	57.23
	Al	3.01	4.45	3.90	3.00	4.70	4.06
	Nb	1.50	1.00	2.11	2.90	3.45	3.93
	Total	100.00	100.00	100.00	100.00	100.00	100.00
	**Spectrum**						
**Ti6Al4V/PBS + albumin**	**Element (wt.%)**	**1**	**2**	**3**		**4**	
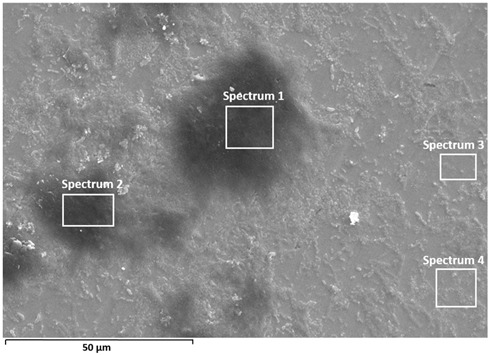	C	53.02	43.84	8.65		8.63	
	N	0.97	1.99	-		-	
	O	20.12	18.06	5.72		7.36	
	Na	2.18	1.96	0.14		0.48	
	Al	1.20	1.72	4.90		4.65	
	P	1.47	0.96	0.01		0.00	
	S	0.30	0.25	-		-	
	Cl	2.07	1.84	0.06		0.20	
	K	0.65	0.39	0.03		0.02	
	Ti	17.23	27.64	79.07		77.40	
	V	0.79	1.35	1.42		1.19	
	Total	100.00	100.00	100.00		100.00	

## Data Availability

The raw data supporting the findings of this study are available from the corresponding author upon reasonable request.
